# Unstable Maternal Environment, Separation Anxiety, and Heightened CO_2_ Sensitivity Induced by Gene-by-Environment Interplay

**DOI:** 10.1371/journal.pone.0018637

**Published:** 2011-04-08

**Authors:** Francesca R. D'Amato, Claudio Zanettini, Valentina Lampis, Roberto Coccurello, Tiziana Pascucci, Rossella Ventura, Stefano Puglisi-Allegra, Chiara A. M. Spatola, Paola Pesenti-Gritti, Diego Oddi, Anna Moles, Marco Battaglia

**Affiliations:** 1 CNR, Cell Biology and Neurobiology Institute, Roma, Italy; 2 Academic Centre for the Study of Behavioural Plasticity, Vita-Salute San Raffaele University, Milan, Italy; 3 Santa Lucia Foundation, European Centre for Brain Research (CERC), Rome, Italy; 4 Department of Psychology, University “La Sapienza”, Rome, Italy; 5 Department of Biomedical Science and Technology, Università dell' Aquila, Coppito, L'Aquila, Italy; 6 Genomnia, Lainate, Italy; 7 Department of Clinical Neuroscience, Istituto Scientifico San Raffaele, Milan, Italy; University of Texas Health Science Center at San Antonio, United States of America

## Abstract

**Background:**

In man, many different events implying childhood separation from caregivers/unstable parental environment are associated with heightened risk for panic disorder in adulthood. Twin data show that the occurrence of such events in childhood contributes to explaining the covariation between separation anxiety disorder, panic, and the related psychobiological trait of CO_2_ hypersensitivity. We hypothesized that early interference with infant-mother interaction could moderate the interspecific trait of response to CO_2_ through genetic control of sensitivity to the environment.

**Methodology:**

Having spent the first 24 hours after birth with their biological mother, outbred NMRI mice were cross-fostered to adoptive mothers for the following 4 post-natal days. They were successively compared to normally-reared individuals for: number of ultrasonic vocalizations during isolation, respiratory physiology responses to normal air (20%O_2_), CO_2_-enriched air (6% CO_2_), hypoxic air (10%O_2_), and avoidance of CO_2_-enriched environments.

**Results:**

Cross-fostered pups showed significantly more ultrasonic vocalizations, more pronounced hyperventilatory responses (larger tidal volume and minute volume increments) to CO_2_-enriched air and heightened aversion towards CO_2_-enriched environments, than normally-reared individuals. Enhanced tidal volume increment response to 6%CO_2_ was present at 16–20, and 75–90 postnatal days, implying the trait's stability. Quantitative genetic analyses of unrelated individuals, sibs and half-sibs, showed that the genetic variance for tidal volume increment during 6%CO_2_ breathing was significantly higher (Bartlett χ = 8.3, p = 0.004) among the cross-fostered than the normally-reared individuals, yielding heritability of 0.37 and 0.21 respectively. These results support a stress-diathesis model whereby the genetic influences underlying the response to 6%CO_2_ increase their contribution in the presence of an environmental adversity. Maternal grooming/licking behaviour, and corticosterone basal levels were similar among cross-fostered and normally-reared individuals.

**Conclusions:**

A mechanism of gene-by-environment interplay connects this form of early perturbation of infant-mother interaction, heightened CO_2_ sensitivity and anxiety. Some non-inferential physiological measurements can enhance animal models of human neurodevelopmental anxiety disorders.

## Introduction

The term ‘separation anxiety’ applies comprehensively to multiple forms of distress reactions displayed by mammals during postnatal development in conjunction with events of separation from a caregiver [1]. Childhood separation anxiety disorder (SAD) - an extreme human manifestation within this interspecies' propensity - predicts heightened risk for panic disorder (PD) in early adulthood [2], and both PD and SAD [3] share a trait of oversensitivity to higher-than-normal CO_2_ concentrations in inhaled air. Relatively specific responses to CO_2_-enriched air mixtures have been described in controlled studies of PD and SAD. These responses consist of both stronger emotional reactions (e.g. panic anxiety), and altered respiratory parameters (i.e., wider tidal volume enhancements and fluctuations, and heightened minute ventilation) [4]–[6], compared to those seen in control subjects.

Whilst PD is part of the DSM-IV anxiety disorders, the bulk of time-honoured data from clinical observation and empirical research indicates that panic attacks should not be equated with fear responses. Clinical panic attacks are typically spontaneous and unpredictable, and characterised by prominent physical symptoms such as dyspnea, rather than by cognitive symptoms [7]. Another physical symptom, frequently reported during spontaneous and CO_2_-provoked panic attacks, is dizziness [8], which may be substantiated in the vestibular dysfunctions often present among people with PD [9]. Endocrinological data contribute to strengthening the view that panic is not a typical emergency fear response. Heightened cortisol levels in spontaneous and CO_2_-provoked attacks [10], [11] have been found to reflect anticipatory anxiety/individual differences in emotionality, rather than the diagnostic category of PD *per se*. Thus, inasmuch as panic attacks occur in the absence of cues of external danger and are triggered by heightened CO_2_ concentrations, they are better seen as inner unconditioned false alarms of biological origin. According to this model, panic attacks derive from a deranged suffocation detector [4] via pathophysiological mechanisms that differ from those underlying general or anticipatory anxiety. Accordingly, most people at the onset of PD are no more anxious/apprehensive/avoidant than people in the general population [12] , and their cortisol levels are within the range of normality [4], [13]. However, after having experienced one or more panic attacks, subjects with PD develop a form of avoidance towards places (such as subways or cinemas) where they believe they will experience dyspnea/panic [14], and also become less explorative towards novel, open spaces, behavioural characteristics collectively named ‘agoraphobia’ [15].

According to twin studies, shared genetic determinants appear to be the major underlying cause of the developmental continuity of childhood SAD into adult PD, and of the association of both disorders with altered sensitivity to CO_2_ [16], [17]. Moreover, a host of events implying unstable parental environment and separation during childhood (encompassing, e.g, parental military service, job relocation, separation, divorce, death, etc.) can account for a significant additional proportion of the covariation between SAD, CO_2_ sensitivity and PD [17]. Thus, in addition to genetic determinants, environmental risk factors affect the liability to these traits, and Ogliari et al. [18] showed that several life events that influence the susceptibility to PD also predict heightened CO_2_ reactivity. There is now initial evidence that genetic and environmental determinants may not simply add, but also interact, to influence human responses to CO_2_. By modelling the effects of life events in young adult twins, Spatola et al. [19] recently found that adversities that take place within the childhood-adolescence window of risk moderate the genetic variance for CO_2_ sensitivity, as assessed by a CO_2_ challenge provocation test. Such a form of gene-by-environment interplay is consistent with a diathesis-stress model, and points towards gene-by-environment interactions [20], [21] that, while rooted in early life, can exert their effect also in early adulthood.

However, the connections between early perturbations of the offspring-caregiver relationships, separation anxiety, panic, and altered respiratory physiology are still to be clarified.

While the study of human subjects is necessarily limited by the ‘natural experiment’ approach, the fact that all mammals show similar physiological responses to heightened CO_2_ concentrations (i.e., hyperventilation and increased arousal/anxiety) can be exploited to disentangle some of the questions that pertain to the human SAD-PD developmental continuum. By capitalizing on physiological responses to heightened CO_2_ concentrations that are relevant to both animal behaviour and the human SAD-PD continuum, one can tackle these questions by fully experimental approaches within the context of animal models [14], [22].

Indeed, higher-than-normal environmental concentrations of CO_2_ constitute an aversive stimulus for many species. In the C. Elegans [23] and the Drosophila [24], [25], CO_2_ elicits innate responses of avoidance. In man, heightened CO_2_ concentrations induce hyperventilation, subjective air hunger and anxiety [18], [26], [27] by activating the ventral medulla and subsequently the pons, midbrain, limbic and paralimbic areas, parahyppocampalgyrus, and the anterior insula [28]. Recent data [29] also show that the amygdala is itself a chemosensor that initiates fear responses under hypercarbia and acidosis.

While all mammals respond similarly to the unconditioned suffocative stimulus of heightened CO_2_ concentration by increasing ventilation, vigilance and eventually by displaying anxious/avoidant behaviour [30], individuals within the same species differ widely from each other in the intensity of these responses, partially due to genetic factors [8], [31], [32]. Moreover, different types of early experiences –including environmental adversities not primarily associated with breathing- may affect the plasticity of the mammalian respiratory control system [33].

To sum up the background of this study, seven interrelated points appear fundamental: 1) SAD and PD are on a developmental and pathophysiological continuum, as SAD often precedes PD, and both conditions are associated with CO_2_ hypersensitivity; 2) hypersensitivity to CO_2_ can be indexed via respiratory parameters and/or exaggerated anxiety responses to heightened CO_2_ concentrations in inhaled air; 3) the phenotypes of hypersensitivity to CO_2_, SAD, and PD share a genetic background; 4) in man, childhood separation from caregivers/unstable parental environment and early life adversities appear to enhance the risk for SAD/PD/CO_2_ hypersensitivity; 5) human responses to heightened CO_2_ concentrations may be in part influenced by complex causal mechanisms, whereby the degree of sensitivity to early environmental adversities appears to be under genetic control; 6) rodents are prone to separation anxiety and respond to heightened CO_2_ concentrations similarly to man, i.e., by incrementing ventilation and arousal/anxiety; 7) also similarly to man, the increase in ventilation under heightened CO_2_ concentrations among rodents yields a degree of interindividual variance, which can be amenable to quantitative genetic estimations.

We speculated that early environmental adversities may moderate a proportion of genetic liability to CO_2_ sensitivity through gene-by-environment interplay mechanisms, and that such mechanisms could be substantiated in man and animals. Inasmuch as CO_2_ sensitivity represents a valid endophenotype [34]–[37] that shares part of the liability with human PD and SAD [17], and since CO_2_ sensitivity is interspecific, animal models of CO_2_ responses can be used as a proxy of a human psychiatric disorder to study gene-environment interplay [38].

To investigate the relationships that link early interference to infant-mother interactions, separation anxiety, and CO_2_ sensitivity, we focused on the ventilatory response to heightened CO_2_ concentrations in outbred mice repeatedly cross-fostered during the first postnatal days. This approach, largely based on respiratory measurements, permits the circumventing of the difficulties that arise from making inferences about an animal's emotional state. Moreover, the laboratory context avoids the gene-by-environment correlations that hamper research on gene-by-environment interplay in man.

By implementing a repeated cross-fostering procedure, we sought to address three main questions that pertain to the human SAD-PD developmental continuum: 1) can this form of perturbation of infant-mother relationship alter the pattern of individual reactivity to inhaled CO_2_? 2) is the alteration in sensitivity to CO_2_ specific and stable? 3) can this type of early manipulation act as an enhancer of individual differences, so that it can reveal mechanisms of genetic control of sensitivity to the environment?

## Materials and Methods

### Animals

NMRI outbred mice (Harlan, Italy) were used in all experiments. Mice were mated when they were twelve weeks old. Mating protocol consisted in housing two females with one male in transparent high temperature polysufone cages (26.7×20.7×14.0 cm) with water and food available *ad libitum*. Room temperature (21±1°C) and a 12∶12 h light dark cycle (lights on at 07.00 a.m.) were kept constant. After 15 days males were removed and pregnant females were isolated in clean cages, and inspected twice a day for live pups. For the first postnatal day (PND0) litters were left with the biological mother.

### Postnatal treatment: Repeated Cross-fostering (RCF) Procedure

The Repeated Cross Fostering procedure (RCF) is a new experimental rearing protocol devised to interfere with infant-mother interaction in the first days of life, thus predisposing offspring to separation anxiety without inducing neglect from caregivers. This was based upon the knowledge that when mouse pups are cross-fostered to adoptive lactating dams, they are usually well accepted and nurtured [39], and on the fact that an adoption procedure carried out in the first postnatal days has a low impact on offspring's HPA functioning [40].

Having spent the first postnatal day (PND0) with the biological mother, on PND1 litters were culled to eight pups (4 males and 4 females) and assigned to experimental Repeated Cross Fostering (RCF), or control (CT) treatment. Unlike the ‘classical’ cross-fostering procedures [41], RCF pups changed caregiver every 24 hours: 4 times in the PND1-PND4 time interval by following a rotation scheme, each dam shifted to 4 different litters and each litter was shifted to 4 different dams (see also Figure S1). The procedure consisted of first removing the mother from the cage, then removing its entire litter, and immediately introducing this litter into the home-cage of a different dam whose pups had just been removed. The RCF pups were then semi-covered with the home-cage bedding of the adoptive mother, which was then reintroduced in the cage and left with this litter for 24 hours. The entire procedure lasted about 30 seconds and took place every day between 10.30 and 11.00 am. This was repeated daily, four times (PND1 to PND4), until reaching the fourth adoptive mother, with which pups remained until weaning (PND0: biological mother, PND1-PND4: adoptive mother 1 to 4- Figure S1). Adoptive dams were lactating females with pups of the same age as fostered litters. This repeated change of caregiver was aimed at interfering with the formation of the infant-mother relationship [42], and to approximate parental instability, a risk factor for internalising disorders, SAD, PD and CO_2_ hypersensitivity in man [17], [43], [44].

Control litters were collected daily and reintroduced to their home-cage, covered with home-cage bedding and had their biological mothers returned within 30 sec, from PND1 to PND4. Animals were weaned when 28 days old and then separated by sex and left in cages with littermates.

Table S1 shows the body weights of RCF and CT individuals during development and in adulthood, as well as their basal body temperature at PND20, measured at a fixed time of day with an infrared body thermometer (153 IRB, Bioseb), in accordance to previously published methods [45].

### Maternal Behaviour

Maternal behaviour was observed daily from PND1 to PND7 by an observer unaware of the litter's status (RCF/CT) in two daily sessions (12.00–12.30 and 16.00–16.30), the first session taking place one hour after the cross fostering procedure on PND1-PND4. Maternal behaviour: a) NURSING, including the arched-back and blanket postures, and b) GP/L: grooming and licking pups [46] was monitored with an instantaneous sampling method (one sampling every 2 min), for a total of 16 sampling points/session. The analyses of maternal behaviour were based on the observation of NURSING and GP/L on 10 litters of RCF, and 8 litters of CT pups.

### Offspring behaviour

Pups' behaviour was evaluated at: a) PND8, by measuring ultrasonic (USVs) distress vocalizations emitted during isolation, and: b) PND10, by measuring the pups' ability to orient towards and approach maternal/home-cage beddings' [47], [48] odour cues (HOMING behaviour, *vide infra*). The assessments of USVs were preceded by transfer of the home-cages into the experimental room at 14.30 of PND8. On PND8, after 1 hour of acclimatization, the mother was removed and transferred into a clean cage, while the offspring was left in the home cage standing on a warm plate set at the temperature of 35,5°C to prevent cooling. Each pup was individually placed for 5 minutes into a beaker containing (i) own-cage bedding (USVs-own) or (ii) clean bedding (USVs-clean) and the vocalizations were recorded. No more than 1 pup/litter/condition was employed and pups were gender-matched for a total of 31 RCF and 44 CT pups. Ultrasonic vocalizations were recorded using an UltraSoundGate Condenser Microphone (CM16, Avisoft Bioacoustics, Berlin, Germany) lowered 1 cm above the top of the isolation beaker containing the pup. The microphone was sensitive to frequencies of 15–180 kHz with a flat frequency response (±6 dB) between 25–140 kHz. It was connected via an UltraSoundGate USB Audio device to a personal computer, where acoustic data were recorded as wav files at 250,000 Hz in 16 bit format. Sound files were transferred to SasLab Pro (version 4.40; AvisoftBioacoutics) for sonographic analysis and a fast Fourier transformation was conducted (512 FFT-length, 100% frame, Hamming window and 75% time window overlap). Spectrograms were produced at 488 Hz of frequency resolution and 0.512 ms of time resolution. To detect ultrasonic vocalizations, an automatic threshold-based algorithm and a hold time mechanism (hold time: 20 ms) were used. Signals below 30 kHz were truncated to reduce background noise to 0 dB. Inaccurate detections were adjusted manually by an experienced user before running the automatic parameter analysis. The total number of vocalizations emitted in 5 minutes was measured.

The pups' orientation towards familiar odorous cues (HOMING behaviour) was evaluated on PND10 in 32 RCF and 36 CT pups. The assessments of HOMING were preceded by transfer of the home-cages into the experimental room at 14.30 of PND10. The amount of time spent in their home-cage bedding-scented versus (i) clean, or (ii) bedding from an alien dam's cage portions of the apparatus was recorded in 5 minute test sessions. The ability of pups to orient towards familiar odorous cues was evaluated in a small apparatus (5×33×10 h cm) with a central plexiglas part (5×5 cm, starting point) that separated (with sliding doors) two differently-scented chambers. One of these was covered with pups' home-cage bedding, whilst the other was covered with (i) clean or (ii) home-cage bedding from an alien dam's cage. After 1 minute of habituation in the central part of the maze, the lateral doors were removed and the pups could move freely in the apparatus. The behaviour of pups in the maze was video-recorded for 5 minutes and the time spent in the different compartments was evaluated thereafter. No more than two pups/litter/condition in a gender-matched design were tested in these two HOMING procedures.

### Basal Corticosterone levels during development

Dams and pups were sacrificed to measure serum corticosterone basal levels collected via trunk blood samples from 10.00 to 11.00 am. Trunk blood samples were collected after decapitation in RCF and CT offspring (RCF = 16, CT = 14, in a gender-matched design) and their corresponding dams as adoptive or biological mothers (RCF dams = 6, CT dams = 5) before weaning (PND 27–28). After blood centrifugation (20 min, 4°C, 4000 rpm) serum samples were stored at −25°C until assay were conducted. Corticosterone levels were measured using commercially available EIA kits (EIA kit Assay Design, sensitivity 27.0 pg/mL). All corticosterone measures were carried out in duplicate.

### Assessments of Face Validity for Human Panic

In order to assess the face validity for human PD/SAD of the current animal model, we capitalised on the following 4 features of human PD, as outlined in the introduction: 1) post-CO_2_ cortisol levels do not differ between subjects at heightened risk for PD and control subjects [10], [11]; 2) after having experienced one or more panic attacks, subjects with PD avoid places where they anticipate experiencing dyspnea [14]; 3) after having experienced panic attacks, subjects with PD become less explorative towards novel, open spaces and develop agoraphobia [15] 4)vestibular dysfunctions are often described among people with PD [9]. These 4 features of human PD were assessed by proxy among RCF vs. CT adult animals in the 4 following experiments:

#### 1. Corticosterone levels after exposition to CO_2_-enriched air Six

The basal serum corticosterone levels obtained from the tail of 6 RCF (2 females 4 males), and 5 CT (2 females, 3 males) individuals (PND70–90) were collected in a litter-balanced design, and compared to corticosterone levels collected in the same individuals by the same tail-cut method after 20 minutes spent an incubator chamber while breathing 6% CO_2_-enriched air. As described above, after blood centrifugation serum samples were stored at -25°C until assay were conducted using the commercially available EIA kits (EIA kit Assay Design). All corticosterone measures were carried out in duplicate.

#### 2. Avoidance of CO_2_-enriched environments

Place avoidance/preference towards a CO_2_-enriched environment was measured in 70–90 day old CT (N = 15) and RCF (N = 11) naïve male mice in a litter-balanced design, in a ‘place conditioning’ apparatus [49] consisting of two differently-cued chambers connected by a central alley. On day 1 (pretest), the mouse was introduced in the central alley and left free to explore the entire apparatus. During the following 8 days (conditioning), each mouse was confined daily (for 20 min) in one of the two chambers, while the apparatus was introduced into an incubator with either room air or 6% CO_2_-enriched air, on alternative days. For each animal, over the 8 training days, one of the two chambers was consistently paired with 6% CO_2_-enriched air and the other one with normal air. Testing was conducted on day 10. Animals were placed in the central alley of the apparatus and left free to explore the chambers for 10 min. Pretest and test sessions were videotaped, and subsequently an experienced observer unaware of the treatment conditions recorded the time (in seconds) spent in the different compartments with dedicated software (Smart, Panlab). Place-preference scores were calculated as: [(the amount of time spent in the CO_2_-paired compartment)/(amount of time spent in both compartments)]×100.

#### 3. Effects of CO_2_ exposure on exploratory behaviour

Animals (78 individuals, 70–90 PND, gender- and litter-matched) for this experiment were first isolated for 24 hrs in a clean cage with a sliding door, with food and water available. Then subjects were exposed either to room air (RCF: n = 21, CT: n = 20) or to a 20% CO_2_-enriched environment (RCF: n = 20, CT: n = 17) for 2 minutes. Immediately thereafter, each animal was allowed to enter a free exploratory apparatus (70×90 cm) connected with the home cage, by leaving the sliding door open for 10 minutes. Each session was video-recorded, and later the percentage of time spent in the centre of the arena (30×50 cm) during exploration of the apparatus was measured by a dedicated software (Smart, Panlab).

#### 4. Evaluation of vestibular function among RCF and CT individuals

Thirty-seven RCF and 32 CT gender and litter-matched individuals (PND70–90) were assessed for their performance at the Rotarod test [50] as a proxy of balance. After training for three sessions in the preceding day, mice had to maintain balance upon an accelerating rotating rod (four trials, from 4 to 40 rpm in 300 seconds), the dependent variable being each subject's latency to fall from the rod [50].

### Respiratory Responses

We took into account the following respiratory parameters: tidal volume (i.e. the volume of air displaced between normal inspiration and expiration,TV), respiratory frequency (i.e., the number of breaths an individual takes per minute, f), and minute volume (MV, which is obtained by multiplying TV by f).

On PNDs 16–20, sixty-four pups were tested for their respiratory responses. Thirty-six RCF pups belonging to 14 litters, and 28 CT pups belonging to 12 litters were tested; each subject was exposed to only 1 air mixture condition (air/6%CO_2_/10%O_2_). For each litter a maximum of 2 pups (one male and one female) were exposed to the same air mixture (air/6%CO_2_/10%O_2_). We used an unrestrained plethysmograph (PLY4211, Buxco Electronics, Sharon CT) carrying two separate Plexiglass chambers of 450 ml, allowing for the parallel assessment of two animals/session.

**Figure 1:**
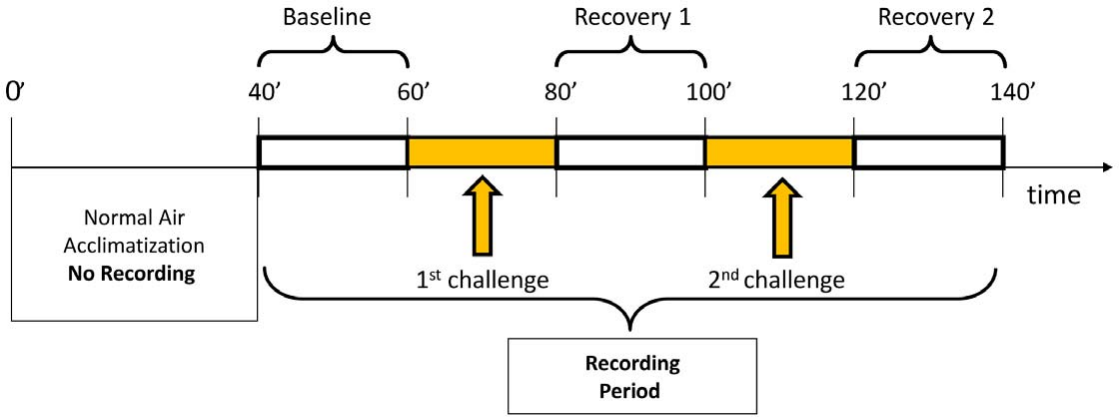
Scheme of the respiratory protocol. During ‘baseline’ and ‘recovery’ periods, subjects inhaled normal air. During ‘challenge’ periods subjects were exposed to one type of air mixture: 6% CO_2_-enriched air, or 10%O_2_ air, or normal air.

Before recording, each subject was closed in the chamber for an acclimatisation of 40 minutes without any air mixture being administered. Subsequently the recording of respiratory parameters started under air condition (baseline) for 20 minutes. Next, the first challenge began: this lasted 20 min and could consist of any of the following three conditions: 1) normal air (20%O_2_); 2) 10%O_2_, 3) 6%CO_2_. A 20 min recovery period (air) followed, then the second challenge (20 min with the same gas mixture as employed in the 1^st^ challenge) took place. A 2nd recovery period (air) of the same duration of the 1^st^ recovery followed, which ended the trial and the recording time. A complete session thus lasted 140 minutes per animal (Figure 1).

Similarly, on PND 75–90, seventy-six adult mice were tested with the same device and procedures, except that only normal air (20%O_2_) or 6%CO_2_ were employed for adults, given the negative results (see results section) with 10%O_2_- and the positive results with 6%CO_2_ stimuli in pups. A with the pups, a maximum of 2 adult subjects (one male and one female) per litter were tested under one air mixture (air/6%CO_2_). Of the 76 adult mice that underwent the respiratory challenges, 33 had previously been exposed to an air/6%CO_2_ challenge as pups: for their challenge in adulthood we used the same air mixture they had been exposed to as pups.

Preliminary tests by general linear models were run to assess whether during the first 20 minutes of pre-challenge baseline recording with air, the TV, f and MV parameters differed between RCF and CT pups and adults, and no significant differences were found. Since TV changes are a major physiological strategy to reduce blood P CO_2_, we capitalized on the mean percentage of TV increment (ΔTV%) from baseline to air/6%CO_2_/10%O_2_ as a reference measure to compare animals' respiratory responses to the air mixtures in the experiments. We also preliminarily tested the effects of: weight, sex, and chamber (there were 2 separate plethysmographic chambers) on the respiratory responses of RCF and CT pup and adult subjects separately, by regression procedures. For adult mice, we also tested the effect of previous participation to the challenge as pups. None of these four predictors influenced the respiratory measurements under air/6%CO_2_/10%O_2_. However, for adult mice, regression of ‘previous participation to the respiratory challenge as pup’ upon the ΔTV% to 6%CO_2_ provided a r = 0.18, F_1,74_ = 2,44, which approached significance: p = 0.12. This variable was therefore added as covariate in the analyses of adult respiratory responses.

The effects of treatment (air vs. 6%CO_2_ vs. 10%O_2_ in pups, and air vs. 6%CO_2_ in adults) and postnatal manipulation (RCF vs. standard rearing in CT) in the two exposure challenges were then tested by repeated-measures ANOVA; upon confirmation of significant main effects, differences among individual means were analyzed with post-hoc Tukey's HSD test.

Since the respiratory outcomes in the first and second challenge were highly correlated for all parameters (mean r = 0.82, p = 0.000001, in pups and adults), for the sake of conciseness we only show the results with the first respiratory challenge in pups and adults.

### Quantitative Genetic Investigation of Individual Differences for CO_2_ Sensitivity

In quantitative genetics, the variance observed for a given phenotype in a group can be partitioned into a proportion attributable to genetic factors, and another proportion attributable to environmental factors, provided that the degree of genetic relatedness among individuals in the study group is known. Thus, depending on circumstances, one can derive from the phenotypic measurements of human monozygotic and dizygotic twin pairs (additive genetic correlation: 1 and 0.5, respectively), or from the phenotypic measurements of unrelated, half-sib and full-sib animals (additive genetic correlation: 0, 0.25 , and 0.5, respectively), the ratio of genetic variance to phenotypic variance or heritability (h^2^) [51]. Gene-by-environment interplay (GXE) mechanisms assume that genetic variance changes as a function of environmental exposure [20], [21]. Likewise, in a typical stress-diathesis GXE model, one may observe that the heritability for the trait under study increases as a function of environmental adversities.

To investigate the nature of the proportion of variance of the respiratory response to CO_2_ associated with RCF, we crossed 8 unrelated naïve sires with 16 unrelated naïve dams in a 1 sire/2 dam breeding design, yielding litters of 8 pups/dam in full-sib/half-sib degrees of relatedness. Eight litters were exposed to the RCF procedure, while the other eight were reared normally, yielding 64 RCF and 64 CT offspring within a full-sib/half-sib (fs/hs) design [51], whereby for each sire both litters were assigned to the RCF- or CT post-natal condition, as appropriate to conduct variance component analysis, nested ANOVA and heritability estimates [51]. Pups in the fs/hs design were assessed only for respiratory phenotypes.

### Animal Care and Statistical Analyses

Unless otherwise specified, all animals took part in only one of the different experiments outlined in this paper, so that they were all naïve individuals. All experiments were conducted under license from the Italian Health Department and in accordance with Italian regulations on the use of research animals (legislation DL 116/92) and NIH guidelines on animal care.

Data were analysed using general linear model approaches, nested ANOVA or variance component analysis, as appropriate.

The variability among dams in providing NURSING and GP/L to fostered (RCF) pups was assessed by two separate ANOVA (Factor: dam) of maternal behaviour collected from PND1 to PND7.

We tested whether the degree of variability in receiving NURSING and GP/L among RCF litters could be assumed as homogeneous by the Levene test (factor : litter).

Similarly, we assessed whether the degree of variability in receiving NURSING and GP/L could be comparable between RCF and CT pups (factor : post-natal treatment) by the Levene test.

Finally, to dissociate the role played by maternal care received by the “final” adoptive mother from PND5 onward from the effects exerted by changes and variation in care received across the 4 cross fostering days (PND1-PND4), we compared the means and variances for NURSING and GP/L in 10 RCF litters by dividing the periods of maternal care into 2 periods of respectively 4 days (PND1–4) and 3 days (PND5–7), as factors.

To ensure sufficient statistical power, the number of subjects in the experiments was determined on the basis of pilot studies carried out in pups and adults. For all figures, bars on histograms indicate standard errors, for all experiments significance was set at p≤0.05.

## Results

### Maternal Behaviour

**Figure 2:**
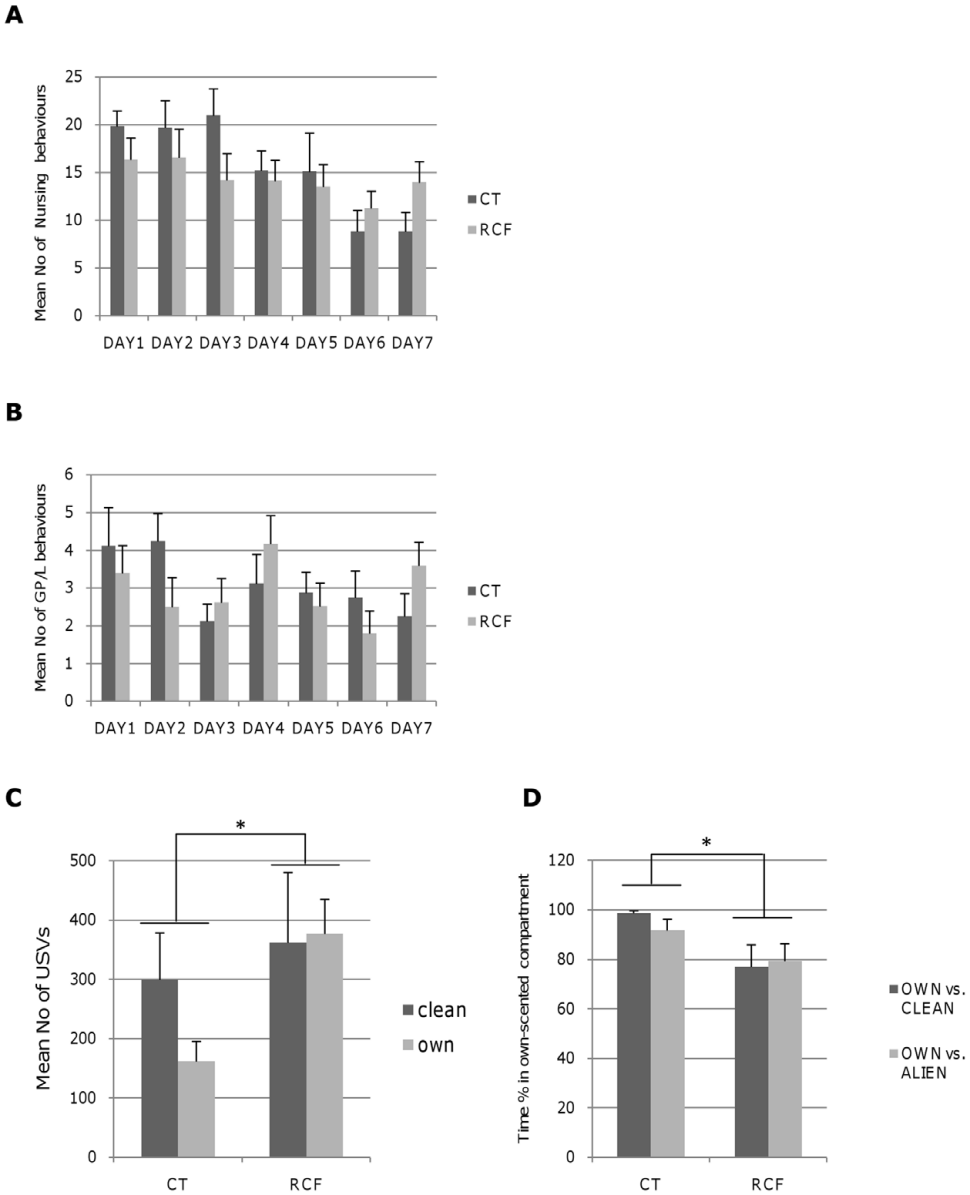
Maternal care and offspring behavioural indices in standard rearing (CT) vs. repeated cross-fostering (RCF) conditions. Sum of 2 daily observations of maternal behaviour: **A**) nursing behaviour, encompassing ‘arch-back’+‘blanket’ postures, and **B**) grooming/licking (GP/L) behaviour towards adoptive (RCF n = 10) and own (CT n = 8) litter, measured during PND1–PND7. Nursing decreased significantly in time, and was comparable in RCF and CT pups across the PND1–PND7 time span. Grooming/licking (GP/L) did not vary significantly in time, and RCF and CT pups received comparable GP/L (see Methods and Results sections for details). Pups' behaviour: **C**) Mean number of ultrasonic vocalisations (USVs) emitted by 8-day old RCF and CT pups. Pups were isolated and exposed for 5′ to fresh clean bedding (clean) and own-cage bedding (own). ANOVA showed that the postnatal treatment (RCF vs. CT) yielded a significant effect (F_1,73_ = 4.24, p = 0.04) while the condition (‘clean’ vs. ‘own’ bedding) did not exert a significant effect (F_1,73_ = 0.84, p = NS); there was no significant postnatal treatment-by-condition effect (F_1,71_ = 1.29, p = NS). **D**) Percentage of time spent during 5 minutes by pups in a compartment containing own-cage vs. fresh clean bedding (own vs. clean), or own-cage vs. an alien dam's bedding (own vs. alien dam). RCF pups spent less time in the compartment with own-cage bedding than controls in both conditions (F_1,64_ = 7.46, p<0.01).

Nursing decreased significantly in time (F_6,119_ = 3.48, p<0.0035, Figure 2A) across the PND1-PND7 time span, but neither post-natal treatment, nor time-by-post-natal treatment yielded significant effects (respectively: F_1,124_ = 0.85 p = NS; F_6,112_ = 1.27 p = NS); consistent with these data, there was no significant difference between RCF and CT mice for weight, measured at different stages of development from PND8 through PND90 (see also Table S1).

Grooming/licking (GP/L) did not vary significantly in time (F_6,119_ = 1.5 p = NS, Figure 2B), and neither post-natal treatment (F_1,124_ = 0.12 p = NS) nor the interaction of post-natal treatment-by-time yielded significant effects (F_6,112_ = 1.33 p = NS).

By ANOVA (factor: dam) we found that NURSING did not differ significantly among dams who took care of the RCF pups (F_9,30_ = 1.45 p = NS) as ‘adoptive mothers’, but GP/L differed significantly among these dams (F_9,30_ = 2.90 p = 0.01). However, when we estimated the differences in variance between 10 RCF litters (estimated across 7 days and 4 different dams/litter) for the amount of received care (factor: litter) by Levene test, we found significant differences neither for NURSING (Levene's _9,60_ = 1.14, p = NS) nor for GP/L (Levene's _9,60_ = 1.46, p = NS). Thus, while there was a certain degree of variability for maternal GP/L towards the fostered pups that was attributable to dams as individuals, the differences in variance of received care among RCF litters from PND1 to PND7 were not significant. This implies that although each RCF litter received care from 4 different dams (i.e., 4 different ‘foster mothers’) in the PND1-PND4 period, the amount of variability of GP/L and NURSING could be assumed as homogeneous among RCF litters.

Similarly, the total variance of NURSING and GP/L did not differ between RCF and CT pups: when we assessed by the Levene test whether the degree of variability in receiving NURSING and GP/L could be comparable between RCF and CT pups (factor: post-natal treatment), we found no significant differences (NURSING Levene's_1,124_ = 2.86, p = NS; GP/L Levene's_1,124_ = 0.53, p = NS). This implies that (inasmuch as the variances of NURSING and GP/L could be assumed as sufficient indicators of postnatal treatment) RCF and CT pups were exposed to similar amounts and variability of postnatal care, the only difference between the two types of postnatal treatment being the intrinsic instability of the caregiver.

Finally, when we compared the means and variances for NURSING and GP/L among RCF pups during the PND1-PND4 period, as opposed to the following PND5-PND7 period (factor: PND1–4 vs. PND5–7), we found that the means and variances for both indexes could be assumed as equal (NURSING: Levene's_1,68_ = 2.43, p = NS; GP/L: Levene's_1,68_ = 0.27, p = NS; NURSING: ANOVA F_1,68_ = 1.86, p = NS; GP/L ANOVA F_1,68_ = 1.03, p = NS). This implies that within the RCF group, NURSING and GP/L were quantitatively similar when pups were exposed to changing caregivers, and in the following first 3 days of stable motherhood.

### Offspring's Behaviour

At PND8, during two different isolation paradigms (conditions: a) ‘clean bedding’ and b) ‘own cage bedding’, see also *methods*) we observed an effect of postnatal treatment upon the isolation distress calls in the form of more USVs emitted by RCF pups. Figure 2C (see also caption) shows that there was no ‘postnatal treatment-by-condition’ interaction. The lower RCF vs. CT difference in isolation distress calls during the ‘clean bedding’ condition (Figure 2C) may indicate a ‘maximum stimulation’ effect induced by the absence of any odour in this specific experimental condition. Consistently, a milder stimulation condition (isolation in ‘own cage bedding’) appeared to unmask the RCF-CT differences more sharply. Sex of subjects did not yield a significant effect, alone or in interaction with the other independent variables.

At PND10, when RCF pups were tested for their preference between own-cage bedding as an alternative to: a) clean bedding, or: b) to an alien dam's bedding (Figure 2D), they showed consistently reduced preference for their own bedding compared to CT pups (Figure 2D). Sex of subjects did not yield a significant effect, alone or in interaction with the other independent variables.

### Corticosterone basal levels in RCF and CT Individuals

The mean (±SE) basal serum concentration of corticosterone did not differ in lactating RCF and CT dams (ng/ml: 45.48±17.79 vs 53.02±11.87 respectively; F_1,9_ = 0.11, p = NS). Likewise, variance components analyses showed that serum concentrations of corticosterone at PND27–28 did not differ amongst RCF (n = 16 subjects belonging to 4 sibships, n/ml 62.70±3.86) vs. CT subjects (n = 14 subjects belonging to 4 sibships, ng/ml 70.27±4.25; fixed: maternal effect F_1,6_ = 1.38, p = 0.28: random: sibship effect F_6,22_ = 1.36, p = 0.27).

### Assessments of Face Validity for Human Panic (1–4)

#### 1 Corticosterone levels after exposition to CO_2_-enriched air

**Figure 3:**
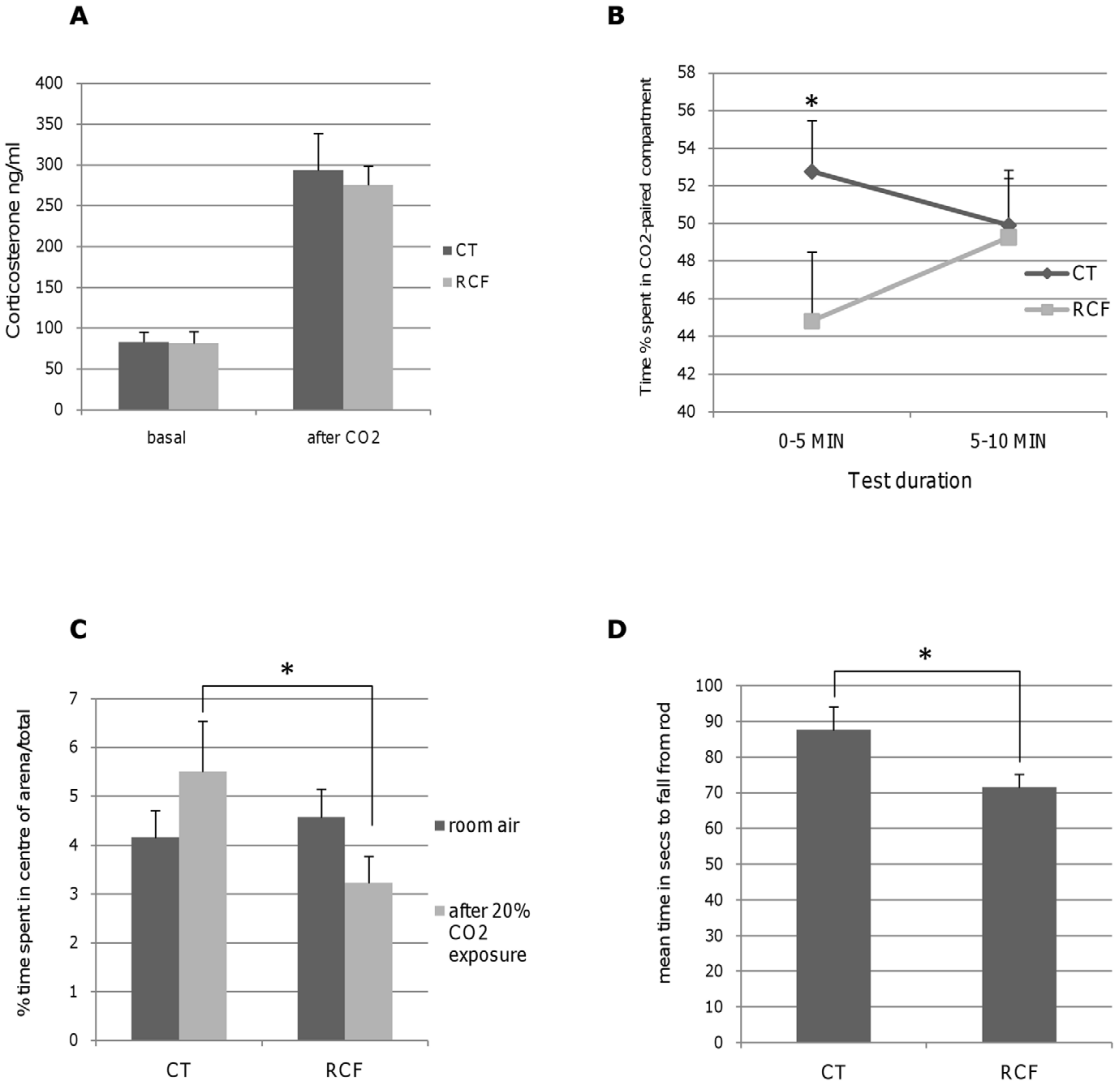
Behavioural and Endocrinological Phenotypes among RCF and CT subjects. **A**: Serum concentration of corticosterone. In both RCF and CT subjects corticosterone was significantly heightened (F_1,9_ = 49.71 p = 0.0001) after 20 minutes of 6% CO_2_ breathing compared to the basal values obtained in room air breathing; neither ‘postnatal treatment’ (RCF vs. CT) nor ‘postnatal treatment-by-air mixtures’ revealed differences for corticosterone serum concentrations (respectively F_1,9_ = 0.2 p = NS, and F_1,7_ = 0.08, p = NS). **B**: Place avoidance/preference towards environments with heightened CO_2_ concentration (6% CO_2_ air mixture): during the first five minutes of the test session, RCF individuals showed significantly higher tendency to avoid the compartment that had been previously paired with 6% CO_2_. (ANOVA-R : postnatal manipulation x time interval effect: F_1,22_ = 4.51, p<0.05, Tukey HSD post-hoc test p<0.02). **C**: Free exploratory test. The percentage of time spent at the centre of an arena was significantly influenced by the interaction of postnatal treatment-by-air mixtures (F_1,74_ = 4.03, p = 0.048) whereby RCF subjects spent significantly less time than CT subjects after exposure to 20% CO_2_. Neither the ‘postnatal treatment’ (RCF/CT), nor the ‘air mixtures’ (normal air/20% CO_2_) variables showed significant effects alone (respectively F_1,76_ = 1.90 p = NS, F_1,76_ = 0.13 p = NS). **D**: Latency to fall from the Rotarod. Analysis of variance showed that the performance at the Rotarod was significantly worse among RCF than CT subjects (F_1,67_ = 5.08 p = 0.03), in that the former showed significantly shorter latency of fall from the rod.

Figure 3A shows that after 20 minutes of 6% CO_2_ breathing the serum concentration of corticosterone was heightened in a similar fashion among RCF and CT subjects.

#### 2 Avoidance of CO_2_-enriched environments

Adult (PND 70–90) RCF mice exposed to a place conditioning protocol displayed an immediate avoidant response to environments associated with heightened CO_2_ concentration, compared to CT mice, as shown in Figure 3B.

#### 3 Free exploratory test after exposition to CO_2_-enriched air

A free exploratory test showed that after exposure to 20% CO_2_, RCF subjects have a significant reduction of the percentage of time spent at the centre of an arena compared to CT subjects (Figure 3C). After being exposed to room air, on the contrary, RCF and CT subjects did not differ significantly for this behaviour. Sex of subjects did not yield a significant effect, alone or in interaction with the other independent variables.

#### 4 Balance test (Rotarod) in RCF and CT individuals

The performance at the Rotarod shown in Figure 3D was significantly worse among RCF than CT subjects, in that the former showed significantly shorter latency of fall from the rod, which suggests impaired balance. Sex of subjects did not yield a significant effect.

### Respiratory Parameters in RCF and CT Individuals

**Figure 4:**
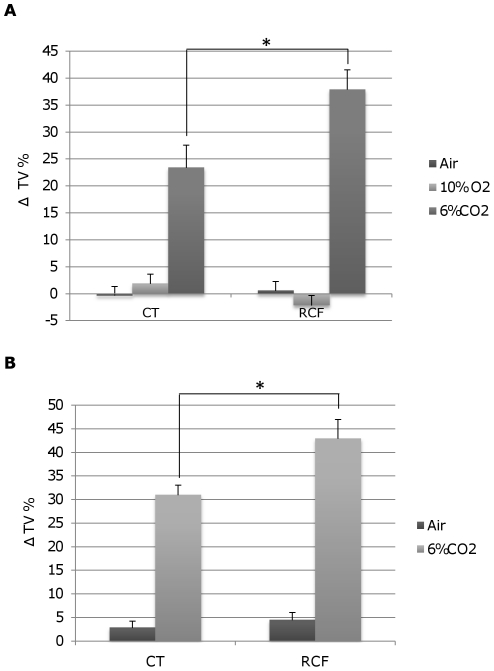
Respiratory responses to air, 10%O_2_, or 6%CO_2_ in CT and RCF subjects at different ages. Percentage of tidal volume changes from baseline (ΔTV%) for: **a**) 16–20 day-old pups in response to normal air, 10% O_2_, or 6% CO_2_. The ANOVA-R carried out on two consecutive respiratory challenges (as depicted in Figure 1) indicated a significant effect of: 1) treatment (type of air mixture): F_2,58_ = 91.30, p = 0.000001, 2) time: F_1,58_ = 4.34, p<0.05, and 3) an interaction effect of postnatal manipulation-by-type of air mixture: F_2,58_ = 9.99, p<0.0002); Tukey HSD post-hoc test p<0.001; **b**) 75–90 day-old adult mice in response to normal air or 6% CO_2_. The ANOVA-R carried out on two consecutive respiratory challenges (as depicted in Figure 1) indicated a significant effect of: 1) treatment (type of air mixture): F_1,71_ = 184.83, p = 0.00001, 2) time: F_1,72_ = 35.12, p = 0.00001 and 3) an interaction effect of postnatal manipulation-by-type of air mixture F_1,71_ = 6.60, p = 0.012). Tukey HSD post-hoc test p<0.001. Sample sizes varied between 9 and 13 animals per group. Only the responses to the first of two consecutive challenges performed for each subject with the same air mixture (air/10% O_2_/6% CO_2_) are shown in Figure 4 for the sake of conciseness.

The RCF procedure was associated with higher mean percent increment of tidal volume from baseline (ΔTV%) during 6%CO_2_. At PND16–20, the RCF pups showed one-and-a-half times the ΔTV% increase shown by CT pups when they were exposed to 6% CO_2_-enriched air mixture, but no difference of ΔTV% in response to hypoxic air (10% O_2_), or normal air, compared to CT pups (Figure 4A). The TV increase determined higher MV amongst RCF than CT pups in response to 6%CO_2_ (mean MV during 1^st^ 6%CO_2_ challenge, respectively: ml/min 53.10±18.22 vs.40.98±12.65, p = 0.009), whereas the mean respiratory frequency (f) did not differ significantly in RCF and CT pups during air, 10%O_2_, or 6%CO_2_ conditions. The RCF procedure also appeared to affect individual sensitivity to 6%CO_2_ in a stable way, in that adult mice (age 75–90 days) that had experienced postnatal RCF, but no other adverse event thereafter, showed higher ΔTV% than CT mice in response to 6%CO_2_ (Figure 4B). For both pups and adult mice, the respiratory response to 6%CO_2_ was more marked during the 1^st^ challenge than during the 2^nd^ challenge, as shown by a significant effect of ‘time’ in the general ANOVA-R models, but differences between RCF and CT animals remained consistent and significant within the first- and second challenge recordings.

### Individual Differences for CO_2_ Responsiveness and Gene-Environment Interplay

Among the unrelated individuals which underwent the respiratory challenges shown in Figure 4, the mean variance of ΔTV% during 6%CO_2_ challenges was 57.75 for CT (n = 8) and 124.58 for RCF (n = 13) pups, and: 113.06 for CT (n = 17) and 224.62 for RCF (n = 19) adult subjects (Figure 4). Thus, the RCF procedure appeared to act not only as an enhancer of the mean physiological response to 6%CO_2_, but it also acted as a trigger to disclose individual differences for the response to CO_2_ amongst unrelated individuals.

On the basis of this datum, and to explore the nature of the proportion of variance of the respiratory response to CO_2_ that appeared to be associated with the RCF procedure, we relied on quantitative genetic analyses of data obtained from the full sib/half-sib (fs/hs) design, as outlined in the Methods section.

Table 1 shows the ΔTV% mean increments during 6%CO_2_ for RCF_fs/hs_ and CT_fs/hs_ subjects at PND 16–20. According to nested ANOVA, this response to 6% CO_2_-enriched air was significantly influenced by both postnatal treatment (RCF vs. CT) and by the degree of genetic relatedness, i.e., a ‘sibship’ factor. Consistent with this result, Variance Component Analysis showed significantly higher genetic variance (Va) for the ΔTV% response to 6% CO_2_-enriched air among RCF_fs/hs_ than CT_fs/hs_ individuals, and almost double heritability, as estimated from half-sibs' correlations [51], for ΔTV% response to 6% CO_2_-enriched air among RCF_fs/hs_ compared to CT_fs/hs_ subjects. The 0.21 heritability we found for ΔTV% among CT_fs/hs_ is close to the 0.24 heritability value reported for TV increase under continuous respiration of 7% CO_2_-enriched air mixtures in normally-reared rats [52]. The significant difference between the RCF_fs/hs_Va and the CT_fs/hs_Va for ΔTV% response to 6% CO_2_-enriched air, and the sizable increase in heritability, indicate the presence of genetic control of sensitivity to the environment [53] evoked by the RCF procedure.

**Table 1:** Tidal Volume percent increment (ΔTV%) in response to 6% CO_2_ in RCF and normally-reared (CT) pups at postnatal day 16–20: Mean Values, Genetic Variance, and Heritability figures estimated from unrelated, half-sib and full-sib individuals.

	CTfs/hs	RCF_fs/hs_
**Mean ΔTV%** response to 6% CO_2_-enriched air	34.79±14.63	42.50±17.43*
**Genetic Variance for ΔTV%** response to 6% CO_2_-enriched air	60.01	125.25†
**Heritability for ΔTV%** response to 6% CO_2_-enriched air	0.21	0.37

Fs = Full sibs; hs = Half-sibs.

*Nested ANOVA: ‘postnatal treatment RCF vs. CT ’ : F_1,112_ = 8.29, p = 0.0048; ‘sibship’: F_14,112_ = 2.17, p = 0.01.

† Bartlett χ^2^ = 8.3, p = 0.004 by Variance component analysis.

Heritability was calculated on the basis of half-sibs'correlations.

## Discussion

Our results show that the respiratory reactivity to CO_2_-enriched air can be modified by a form of environmental adversity that is not primarily associated with breathing, namely repeated cross-fostering during the first postnatal days. While the RCF protocol used in this study did not interfere with the pups' normal development, as shown by comparable weights and body temperatures in RCF and CT mice, it may have interfered with the formation of infant-mother selective bond. Accordingly, our behavioural data show that while the RCF procedure did not evoke a response of neglect from adoptive mothers, it induced measurable behavioural distress -such as higher number of separation calls, typically interpreted as sign of separation anxiety [54]- amongst RCF pups. The RCF also possibly altered the ability to orient and approach maternal cues among these pups. More importantly for the aims of this investigation, the RCF procedure appeared to impact upon sensitivity to 6% CO_2_-enriched air selectively (as responses to 10%O_2_ and normal air were unaffected by the RCF), and stably from childhood into early adulthood (as responses to 6% CO_2_ were similar in pups and in adult RCF subjects). Such specificity of effect conforms with the notion that the regulatory mechanisms of hypercapnic and hypoxic ventilatory responses are functionally separated and genetically dissociated in mice [31]. Moreover, these results point towards an effect of RCF upon the central, more than the peripheral chemoceptors, since the former are much more sensitive to changes in P CO_2_ (monitored as [H^+^]), than changes in P O_2_. The ‘classical’ maternal separation protocol (e.g., 3 hours/day for 10 consecutive days) has been reported to influence the respiratory responses in rats [55]. However these effects are less specific, in that both the responses to hypoxia and CO_2_ are altered, and less straightforward to interpret, as opposite patterns of ventilatory response heightened CO_2_ have been observed in male and female rats [54]. Unlike what we observed in our RCF mice via the corticosterone data, in rats the 3 hours/day for 10 consecutive days procedure of maternal separation enhances the basal hypothalamic-pituitary-adrenal axis function [56].

The RCF procedure appeared to act not only as an enhancer of the mean physiological response to CO_2_. It also acted as a trigger to disclose individual differences for the predisposition to vary the response to CO_2_. Our data show significant differences in genetic variance and in heritability between RCF and CT subjects. This indicates that mechanisms of genetic control of sensitivity to the environment are operant here, in the absence of the gene-environment correlations that often complicate the interpretation of heritability variation in man [20], [57]. In other words, the RCF procedure brought about a proportion of diversity for CO_2_ sensitivity that was ultimately attributable to genetic effects. One may speculate on variations in gene expressions as one likely molecular explanation of our quantitative genetics results, and genomics approaches would now be needed to further explore this paradigm. The results of molecular genetic analyses in this mouse model of separation anxiety could in turn kindle new molecular genetic approaches to human PD and SAD.

Turning to behavioural variables, while the separation calls in RCF and CT pups showed that our procedure evoked more separation anxiety amongst the former [54], it is tempting to relate the avoidance towards CO_2_-enriched environments shown by RCF mice to the avoidant/escape behaviour that people with PD display towards crowded, closed environments, where they fear they might experience smothering sensations and panic [14]. Likewise, previous exposure to heightened CO_2_ concentrations reduced free exploratory behaviour significantly more among the RCF than the CT subjects.

Two further findings appear to establish a parallel between the RCF mice and humans at heightened risk for the SAD-PD continuum. First, we found similar post-CO_2_ corticosterone concentrations among RCF and CT mice, and cortisol levels following spontaneous and CO_2_-provoked attacks do not seem to bear strongly upon the diagnosis of PD in man [4], [10], [11], [13]. Second, RCF mice performed significantly worse than CT at the Rotarod. Consistent with this finding, vestibular dysfunctions are found more often among people with PD than among healthy controls [9], with dizziness being frequently reported in spontaneous and CO_2_-provoked panic attacks in man [8]. On the other hand, we did not find significant sex-related differences in our respiratory and behavioural tests. This is a possibly relevant discrepancy compared to human data, whereby women typically respond more than men to CO_2_ stimulation and have higher prevalence of PD [8].

In conclusion, inasmuch as the genetic determinants that promote overreaction to heightened CO_2_ concentrations and naturally-occurring panic attacks in man coincide [16], the adoption of objective respiratory responses, more than the inferential assessment of emotionality or behaviour, is a viable strategy for laboratory animal models of human PD. By these same strategies the developmental pathways of continuity from childhood separation anxiety into adult panic disorder, and the association of both conditions with altered sensitivity to CO_2_, can be further clarified, and GXE mechanisms explored in man and animal. Animal laboratory investigations of the mechanisms by which environmental adversities -including childhood unstable parental environment/separation from caregivers- and genetic factors impinge upon CO_2_ sensitivity can set a new basis for future, better-tailored genetic approaches to human neurodevelopmental anxiety disorders [20], [58]. Our findings further support the investigation of the precise causal mechanisms that connect environmental adversities occurring in sensitive periods of development to health status in childhood and early adulthood [59].

## Supporting Information

Figure S1 Scheme of the RCF procedure.
(DOC)

Table S1 Body Weight and Temperature of RCF and CT pups at different developmental stages.
(DOC)
