# Supplementary figures and images for: Unstable Maternal Environment, Separation Anxiety, and Heightened CO2 Sensitivity Induced by Gene-by-Environment Interplay
Source: PLoS One. 2011 Apr 8;6(4):e18637. doi: 10.1371/journal.pone.0018637 (PMC3072999; doi:10.1371/journal.pone.0018637)

**Supporting Information**

**Figure S1.**

**
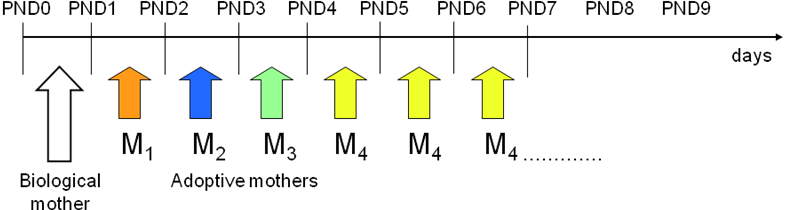
**

Supplement: Figure S1 — Scheme of the RCF procedure. (DOC) [file pone.0018637.s001.doc]
