# Supplementary material for: Unstable Maternal Environment, Separation Anxiety, and Heightened CO2 Sensitivity Induced by Gene-by-Environment Interplay
Source: PLoS One. 2011 Apr 8;6(4):e18637. doi: 10.1371/journal.pone.0018637 (PMC3072999; doi:10.1371/journal.pone.0018637)
Supplement: Table S1 — Body Weight and Temperature of RCF and CT pups at different developmental stages. (DOC) [file pone.0018637.s002.doc]

**Supporting Information.**

**Table S1.**

| Developmental  Parameters | BODY WEIGHT | | | TEMPERATURE |
| --- | --- | --- | --- | --- |
|  | PND 8 (N) | PND 16-20 (N) | PND 75-90 (N) | PND 20 (N) |
| CT females | 5.78 + 0.10 (20) | 11.66 + 0.59 (14) | 32.69 + 2.24 (17) | 37.47 + 0.21 (44) |
| CT males | 5.73 + 0.11 (31) | 11.26 + 0.55 (14) | 45.03 + 1.12 (20) |  |
| RCF females | 5.66 + 0.07 (23) | 10.12 + 0.65 (19) | 31.98 + 1.67 (22) | 37.21 + 0.30 (36) |
| RCF males | 5.63 + 0.06 (26) | 10.19 + 0.75 (17) | 41.64 + 0.93 (17) |  |
| F (df)  post-natal treatment | 1.55 (1,96)  ns | 2.38 (1,60)  ns | 1.69 (1,72)  ns | 0.57 (1,78)  ns |
| F (df)  Sex | 0.17 (1,96)  ns | 0.42 (1,60)  ns | 48.65 (1,72)  p<0.0001 |  |
| F (df)  post-natal treatment x sex | 0.03 (1,96)  ns | 0.05 (1,60)  ns | 0.72 (1,72)  ns |  |
